# A rare entity: Sympathetic ophthalmia presumably after blunt trauma to the phthisical eye and optical coherence tomography angiography metrics to monitor response to treatment

**DOI:** 10.1002/ccr3.2597

**Published:** 2019-12-09

**Authors:** Anadi Khatri, Satish Timalsena, Bal Kumar Khatri, Muna Kharel, Sudhir Gautam, Jeena Gurung, Santosh Kafle

**Affiliations:** ^1^ Birat Eye Hospital Biratnagar Nepal; ^2^ Department of Ophthamology Nepalese Army Institute of Health Sciences Kathmandu Nepal; ^3^ Department of Pathology Birat Medical College and Teaching Hospital Biratnagar Nepal

**Keywords:** angiography, autoantigens, imaging, ocular wounds, optical coherence tomography and angiography, phthisis, sympathetic ophthalmia, uveitis

## Abstract

A blunt trauma to a phthisical eye may elicit sympathetic ophthalmia. Non invasive imaging such as use of optical coherence tomography and angiography metrics of the retinal and choroidal vasculature can help monitor response to the treatment.

## Graphical abstract

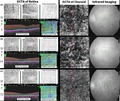

## 1 INTRODUCTION

Sympathetic ophthalmia (SO) is one of the most devastating ocular pathologies. It is elicited by penetrating injury to the eye.

Here, we report an unusual case of sympathetic ophthalmia presumably elicited by trauma to a phthisical eye. We also propose the use of optical coherence tomography (OCT) and angiography (OCTA) metrics to evaluate and monitor response.

Sympathetic ophthalmia is one of the most devastating ocular pathologies. It is elicited by penetrating injury to the eye which could be secondary to an iatrogenic or a noniatrogenic trauma.^1^, ^2^ It is also regarded as one of the best examples of autoimmunity, where the hidden antigens are expressed secondary to an insult. As a result of failure to recognize the “hidden” self‐antigen, a devastating cascade of immunological reaction against the tissues begins.^3^ Here, we report an unusual case of sympathetic ophthalmia elicited by trauma to a phthisical eye.

## 2 CASE

A 38‐year‐old man presented with redness and progressive painless diminution of vision in the left eye (LE) for 5 days. Patient denied any discharge or trauma to the same eye but gave a history of blunt trauma to the right eye (RE) 31 days back with a bamboo stick. His RE was phthisical following penetrating trauma 17 years back inflicted by metal wire. He had received treatment at the regional eye center after which a gradual shrinkage of the eye occurred over the years with the loss of perception of light. However, documentations were not available.

**Figure 1:**
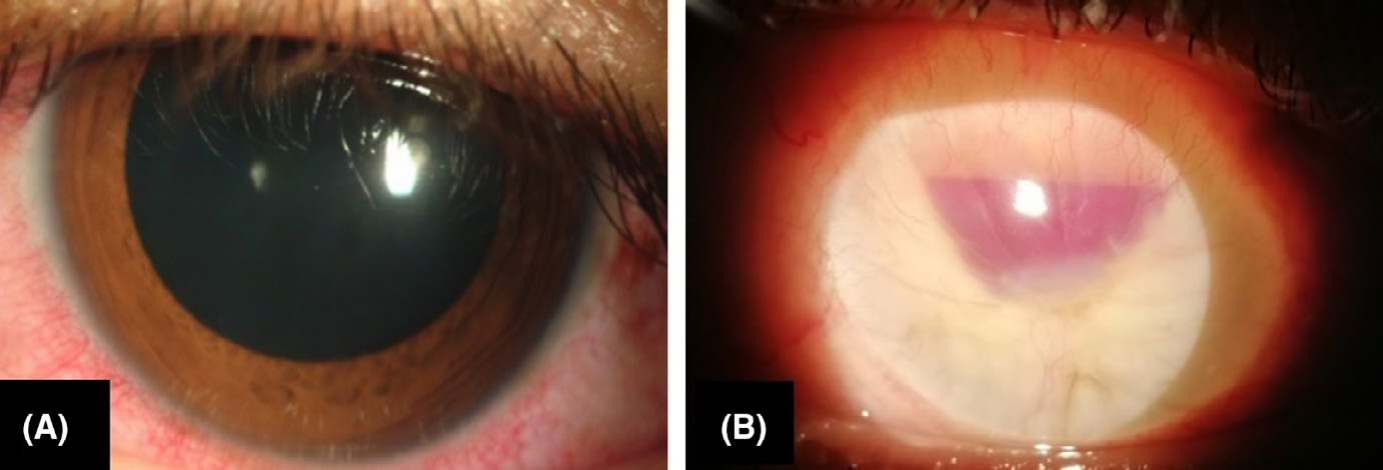
A, The sympathizing eye with circumciliary congestion and uveitis. B, The exciting eye—which is phthisical. Note the blood column behind the disfigured cornea

On examination of the LE, the best‐corrected visual acuity (BCVA) was hand movement with accurate identification of projections. The conjunctiva was congested with circumciliary congestion (Figure 1A). There was a presence of mutton fat keratic precipitates and anterior chamber cellular reactivity (ACR) of 4 + as per standardization of uveitis nomenclauture (SUN) classification.^4^ Posterior segment evaluation revealed a vitreous haze of grade (2+) with the cellular activity of (3+) and disk edema(DE) as per Nussenblat et al's classification.^5^ A shallow macular detachment with a radius of approximately two disk diameters from the fovea was noted.

Clinical examination of his right eye revealed a phthisical eye with localized subconjunctival hemorrhage inferioly, redness around the disfigured cornea, and presence of blood behind it. The patient complained of a dull aching pain in the eye since the blunt trauma (Figure 1B).

**Figure 2:**
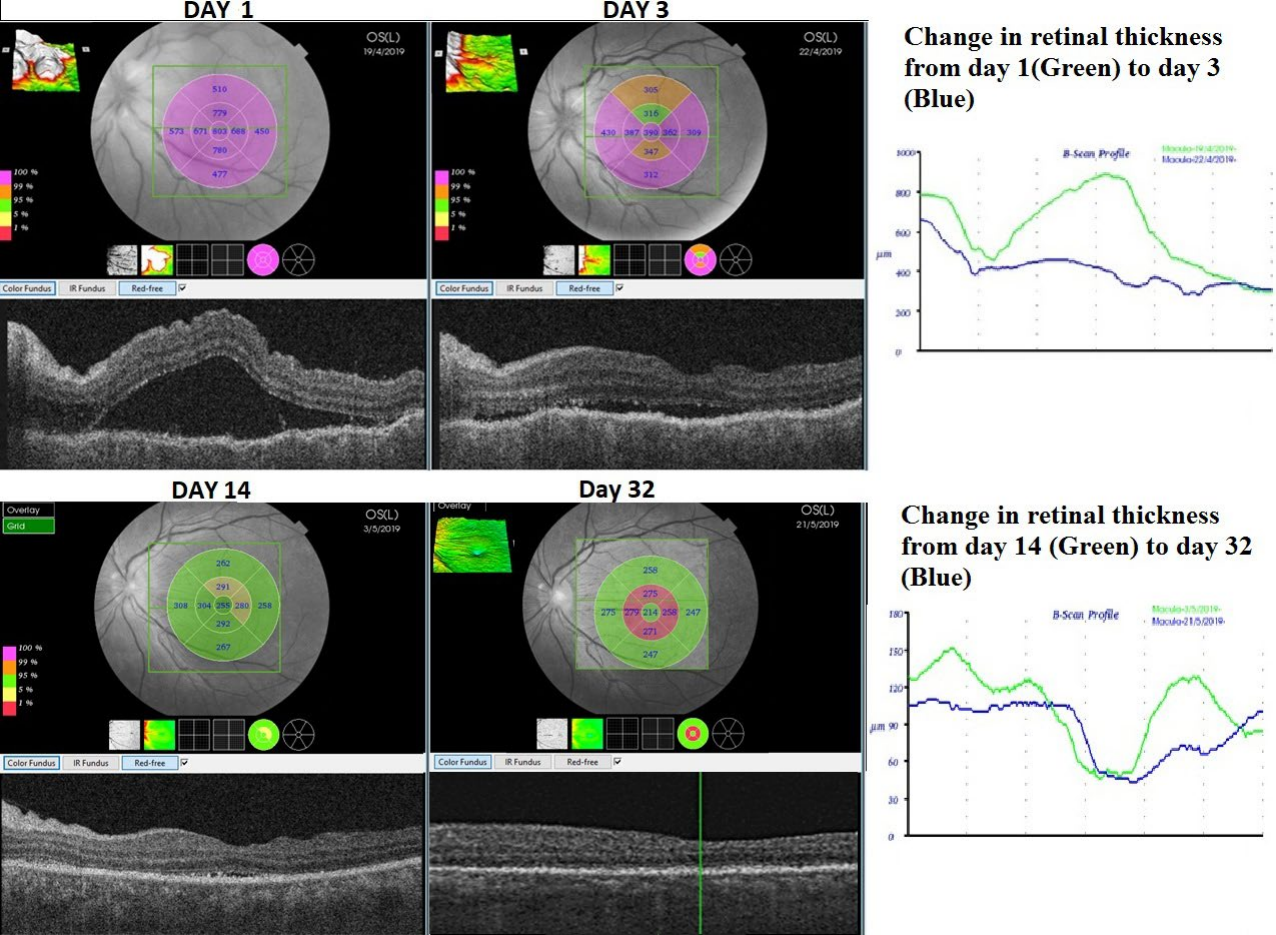
Optical coherence tomography(OCT) findings from day 1 to day 32 after initiation of treatment with steroids and antimetabolites. The decrease in the retinal thickness and subsiding to the subretinal fluid is remarkable and was congruent with vision gain. Note that on day 1, the fovea is decentered as the patient had poor fixation

**Figure 3:**
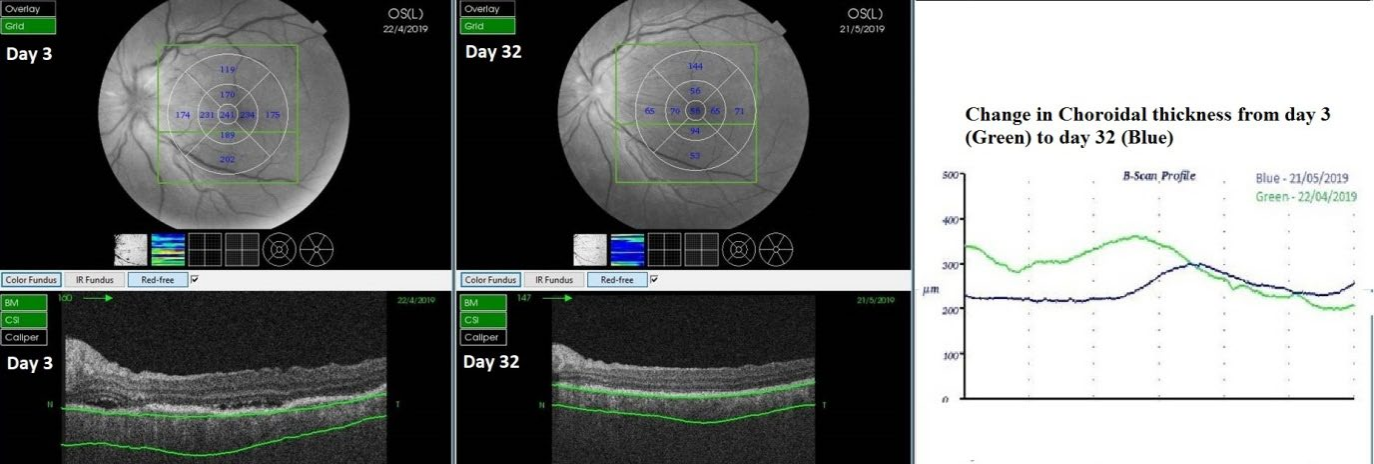
The change in choroidal thickness over 30 d during deep range imaging (DRI) after medication. The mean subfoveolar choroidal thickness was 376 microns during the acute phase which decreased to 234 microns on day 30 following treatment which coincided with good recovery of the vision and subsiding of the serous retinal detachment. Note again that on day 1, the fovea is decentered as the patient had poor fixation

**Figure 4:**
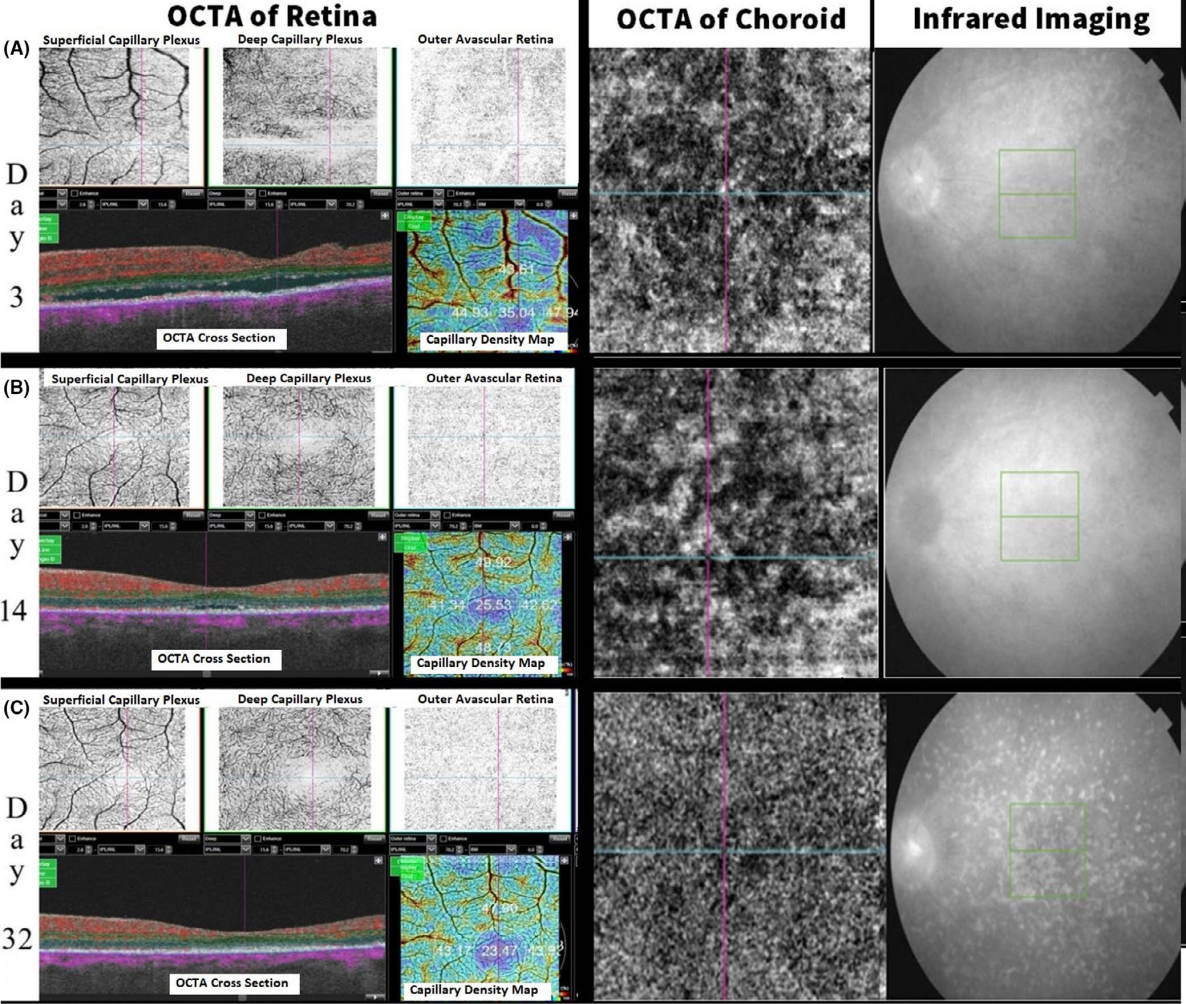
Optical coherence tomography angiography (OCTA) analysis over a period of 32 d after treatment. Vascular plexuses looked disorganized with foveal avascular zone looks indistinct during the acute phase. Note that on day 1, the fovea is decentered as the patient had poor fixation. Following the treatment, both the vascular and the FAZ architecture started becoming organized. The choroidal vascular presented as pockets of flow voids initially and regained its granular looking pattern after initiation of treatment. Note the development of the mottled appearance of the fundus in infrared imaging presumably due to atypical Dalen Fuch's nodules. This remodeling continued even weeks after the retinal and the choroidal thickness became normal and stabalized. Visual acuity of the eye improved during this period

**Figure 5:**
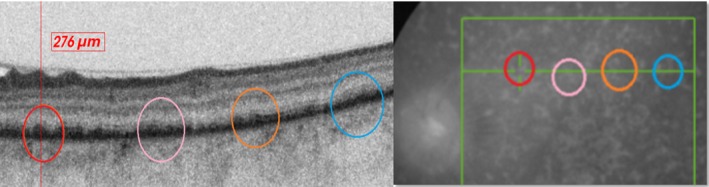
The mottling in the infrared images corresponded to hyperreflective “clumps” inbetween/involving the pigment epithelium and Bruch's membrane. They could represent Dalen Fuchs, chorioretinal adhesions, or active inflammation which would need histological confirmation

The patient was advised for fundus fluorescence angiography (FFA) but had an allergic reaction in test dose, and hence it was not done. An OCTA—(Topcon Medical Systems‐Triton DRI PLUS SS‐OCT) revealed separation of the neurosensory layer with an accumulation of subretinal fluid (SRF, Figure 2). Deep range imaging (DRI) of the choroid showed distorted choroidal architecture with thickening and dilated vascular artefacts (Figure 3). OCTA of the choroidal vasculature revealed pockets of flow voids, and infrared imaging showed development of retinal mottling (Figures 4 and 5). OCTA of the retina revealed distorted capillary plexuses in both the deep and superficial layers with increased vascular density causing shrinkage of the foveolar avascular zone (FAZ; Figure 4).

Laboratory investigations were normal, and after excluding other possible conditions, a diagnosis of sympathetic ophthalmia was considered. The patient was started on oral prednisolone of 120 mg once daily 2 mg/kg/d (bodyweight 57 kg) along with azathioprine 75 mg divided into two dosages along with topical steroids and cycloplegic agents and was advised to follow‐up in 2‐3 days.

On the first follow‐up (day 3 after initiating medication), vision in LE improved to 1/60. ACR was 3+. Vitreous haze had decreased, and OCT revealed a settling neurosensory detachment (NSD) of the retina. On subsequent follow‐ups at days 7, 14, and 32, BCVA continued to improve and stabilized at 6/9 with complete resolution of uveitis and NSD and DE associated with it (Figure 2).

With medication, as the BCVA improved, anatomic recovery was notable in all imaging modalities. Retinal edema and SRF gradually disappeared, choroidal thickening and vascular architecture returned to normalcy with decreased thickness, while angiographically capillary plexuses became more organized, and the FAZ was gradually restored over the course of recovery (Figures 2, 3, 4). The choroidal flow voids that were eventually present as pockets slowly started to disapper and normalized into more granular pattern of vascular pattern in OCTA (Figure 4). In infrared imaging of the fundus, retina gained a prominent mottled appearance after treatment. On analysis of these mottled artefacts on OCT, they represented bumps involving the retinal pigment epithelium (RPE) and Bruch's membrane causing disruptions in ellipsoid which is typical of Dalen Fuch's (D‐F) nodules (Figure 5).

Steroids were tapered, but the patient was continued in azathioprine. The subconjunctival hemorrhage in the phthisical eye had resolved, but blood was still seen behind the disfigured cornea at his last follow‐up.

## 3 DISCUSSION

In this report, we describe an atypical case of SO after blunt trauma to a phthisical eye which endured a penetrating injury 17 years back. Although we were not able to perform the immunological or histopathological study, SO being a more of a clinical entity, our diagnosis was based on history and clinical presentation. An alternate diagnosis which could mimic similar conditions such as Vogt‐Kayanagi‐Harada (VKH) syndrome was ruled out specially given the history of penetrating trauma to the right eye.^5^, ^6^

This was further supported by the findings from the noninvasive multimodality imaging.

The case presented to us in a rather interesting fashion given that the patient was asymptomatic for 17 years in LE following penetrating trauma. This is not atypical given that there have been reports of SO occurring antecedent to penetrating injury ranging from certain weeks to several decades.^1^, ^6^, ^7^, ^8^

Although the controversy does exist that the presentation may not be directly related to the blunt trauma and could be a coincidence, we highly doubt it given that the problem did not exist until the blunt trauma.

The more questionable event is the initiation of SO in LE 3‐4 weeks following blunt trauma to the phthisical RE. Bechrakis et al postulated that a penetrating trauma may not be required for development of SO and damage to certain uveal tissues may itself be sufficient to trigger an immune response.^9^

We were not able to find any literature on SO resulting from a blunt trauma to a phthisical eye, but rare instances where sympathetic ophthalmia has occurred even without any history of penetrating trauma to the eye have been documented. 3, 9

We postulate a theory that a phthisical eye as a result of penetrating injury if subjected to certain blunt trauma may elicit sympathetic ophthalmia. The disorganized blood ocular barrier, vascular permeability, and presence of inflammation could theoretically promote and present hidden uveal antigen to immune cells—hence inflicting a trigger and a response.

Furthermore, studies on histopathological sections of phthisical eyes have demonstrated that certain uveal tissues containing melanocytes continue to demonstrate reactive proliferations—especially in ciliary body epithelium and retinal pigment epithelium.^12^ Melanin antigens along with various other antigens—retinal soluble antigen(S‐Antigen) have both been studied and although not conclusive—have been thought to induce CD4+ cells mediated inflammation with the primary target being uveal tissues.^11^

We suspect similar tissue remnants in a phthisical eye if again disturbed by any insult could elicit sympathetic ophthalmia as in this case.

Fluorescein angiography is very helpful for diagnosing the condition, but it was avoided when the patient started complaining of possible allergic reaction. Although the controversy regarding routine test for allergy and the amount of chemical ingredient to elicit a reaction exist, we avoided this test as a safety precaution.^12^, ^13^, ^14^Noninvasive multimodal imaging studies have been long used to determine the course and response to treatment for SO. OCT findings denoting the resolution of the NSD and decrease in the choroidal thickening has been described in the literature as favorable signs of healing. This is most of the time associated with functional gain with improvement in visual acuity.^9^, ^10^ These descriptions were in agreement with our case as well.

Many studies have mentioned the use of choroidal thickness as a parameter to monitor response for the treatment.^15^, ^16^, ^17^ Although most reports are based on enhanced depth imaging (EDI), we used DRI, and our findings were similar. The sympathizing eye had choroidal thickening with increased vascular artefacts at acute phase which decreased and normalized after treatment. OCTA of the choroidal vascular revealed flow void pockets initially at inflammatory stage, and this normalized over time into typical granular pattern after initiation of the treatment. Similar findings have been reported by Brar et al, where they have mentioned the use of widefield montage OCTA and detected similar areas of flow voids in the choriocapillaris in a case of sympathetic ophthalmia. They have concluded that these areas could likely be due to ischemia which could be used as an noninvasive anatomic marker to monitor treatment response. We were able to confirm this similar finding on a 3 × 3 mm grid. Similar mentions on choroidal vasculature has also been mentioned in cases of VKH and has been mentioned that they are more pronounced during the acute stage of the disease.^18^

Infrared imaging of the retina and choroid revealed a mottled appearance of the fundus after normalization of the retinal and choroidal thickness. On evaluation of these spots on OCT cross sections, they revealed bumps in the RPE and Bruch's membrane with some causing disorganization of the ellipsoid layer. Such findings in OCT have been described by Muakkassa^19^ as typical of D‐F nodule and could represent same in our case. Varghese et al^20^ have also mentioned D‐F nodule again as small nodules seen on OCT. Dell'Arti et al^21^ have also mentioned dome‐shaped choroidal lesions extending up to the inner nuclear layer and interrupting both Bruch's membrane and RPE whch are typical of type 3 D‐F nodule and confirmed using indocyanine green. However, typical D‐F nodule are generally discrete, nodular, and few in numbers. In VKH, it is most often found in the periphery, while in the SO, they are mostly distributed in the midperiphery.^22^In our case, these lesions did not agree with these classical distribution but were instead mainly concentrated in the posterior pole and midperiphery region. Murugan et al^23^ have highlited that sometimes atrophic lesions seen in the convalescent stage can produce similar lesions which erroneously may be confused with D‐F. Histopathologically, such nodules usually reveal focal RPE loss with chorioretinal adhesions. However, they also conclude that even these erroneous lesions are mostly found in the peripheral retina which is again not congruent with our finding. To the best of our knowledge, there is no previous peer‐reviewed indexed literature on the use of infrared imaging and reporting of such lesions and thus this report opens new doors for investigation to confirm the findings—of whether these are atypical D‐F nodule/chorioretinal adhesions or even a sign of an active choroidal inflammation which may previously have been missed by other imaging modalities.

In addition, we also performed OCTA in our patient during the acute, recovery, and stable phase to monitor the response of treatment. OCTA revealed disruption in capillary plexus architecture in both deep and superficial layers with crowding of the FAZ by perifoveal vessels. Following treatment, the capillary plexus started regaining its normal architecture, and the FAZ also started reestablishing. This continued even after weeks after the retina and the choroid had returned to normalcy, and functional improvement in visual acuity continued. Few mentions on OCTA findings in SO have been mentioned, where the authors have described areas of flow voids in widefield montages which recovered after treatment.^24^ However, our description is based only on the findings of the central macular region of 3 × 3. We conclude that trauma to a phthisical eye may elicit SO which if recognized and treated in time and can yield good visual recover. OCTA metrics such as remodeling and recovery of the architecture of the CP and FAZ may also serve as a good indicator in evaluating and monitoring response and planning further management.

## CONFLICT OF INTEREST

We declare no competing interest.

Consent: Written consent was taken from the patient, and highest measures have been taken to ensure that the confidentiality on any information that may reveal patient identity has been maintained.

## AUTHOR CONTRIBUTON

AK, ST, BKK, SG, and JG: were directly involved in management of the case. AK and MK: were involved in preparation and verification of the manuscript and analysis of the images. SK: was involved in providing pathological aspect of the condition. The final manuscript was verified and agreed upon by all the authors.

All authors: made substantial contributions to conception and design, acquisition of data, or analysis and interpretation of data; took part in drafting the article or revising it critically for important intellectual content; gave final approval of the version to be published, and agree to be accountable for all aspects of the work.

## ETHICAL APPROVAL

This is a case report and does not require a review from institution review board of Birat Eye Hospital, Biratnagar, Nepal.
